# Prize Essay on “Diseases of Children”

**Published:** 1871-10

**Authors:** 


### Prize Essay on "Diseases of Children."

The President of the Medical Society of the County of New York, Dr. Abraham Jacobi, has placed in the hands of its Treasurer foui hundred dollars ($400), to be awarded for the best essay on "A History of the Diseases of Infancy and Childhood in the United States, and of their Pathology and Therapeutics." Competitors will send their essays in English, with motto attached, and the name and address of the writer, with the same motto, in a sealed envelope, to the present Secretary of the Society, Dr. Alfred E. M. Purdy, 123 East Thirty-Eighth Street, New York, on or before January 1st, 1873. The Committee are authorized by the Society to withhold the prize if the essays submitted should not merit it.

AUSTIN FLINT, M.D.,
ERNEST KRACKOWIZER, M.D., } Committee.
EDWARD S. DUDSTER, M.D.,

